# Supplementary material for: DEFECTIVE ENDOSPERM-D1 (Dee-D1) is crucial for endosperm development in hexaploid wheat
Source: Commun Biol. 2020 Dec 23;3:791. doi: 10.1038/s42003-020-01509-9 (PMC7758331; doi:10.1038/s42003-020-01509-9)
Supplement: Supplementary file 2 — Supplementary information [file 42003_2020_1509_MOESM2_ESM.pdf]

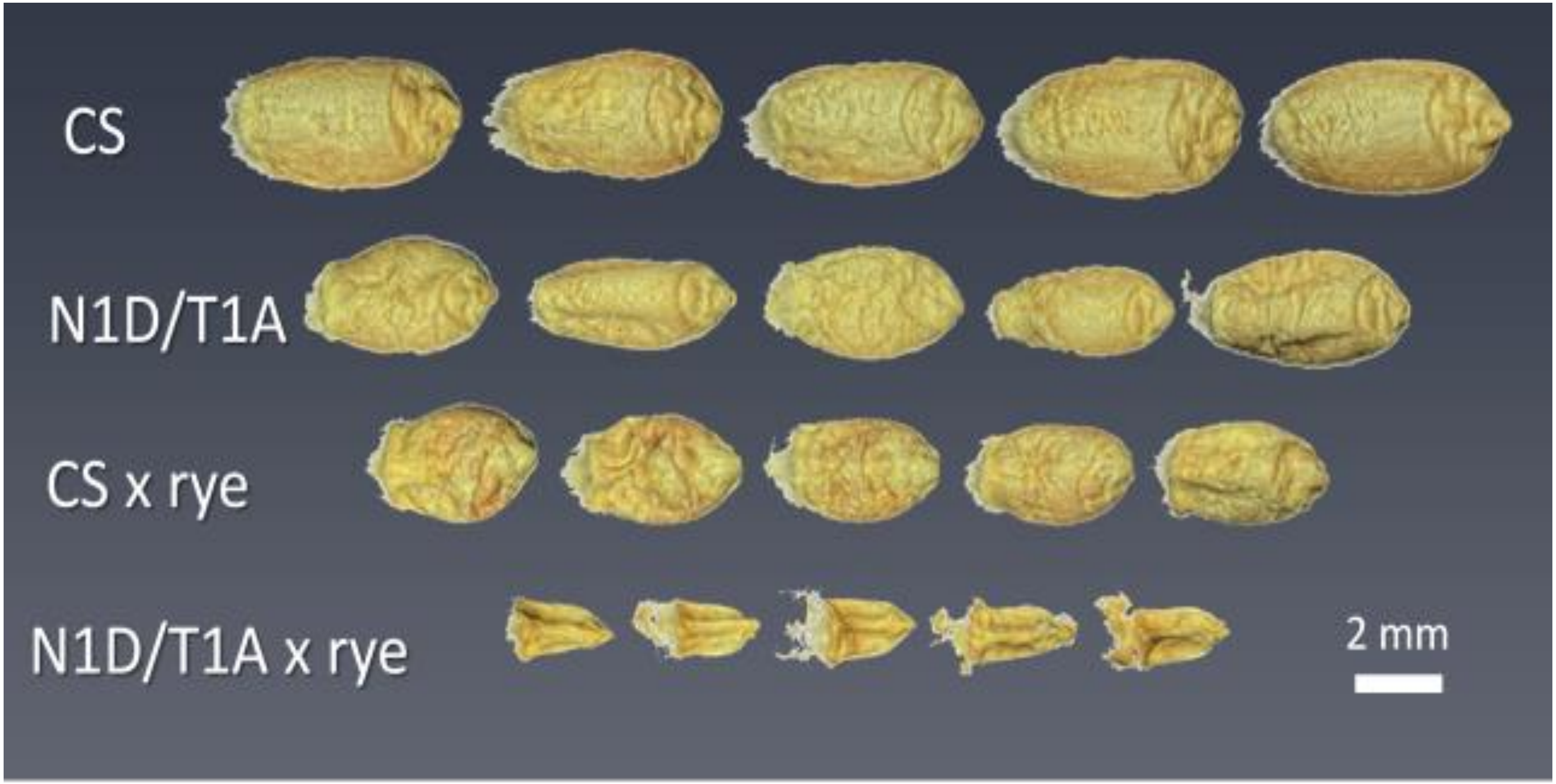

Supplementary Figure 1: Examples of mature grains of hexaploid wheat and crosses (field experiment, 2015).

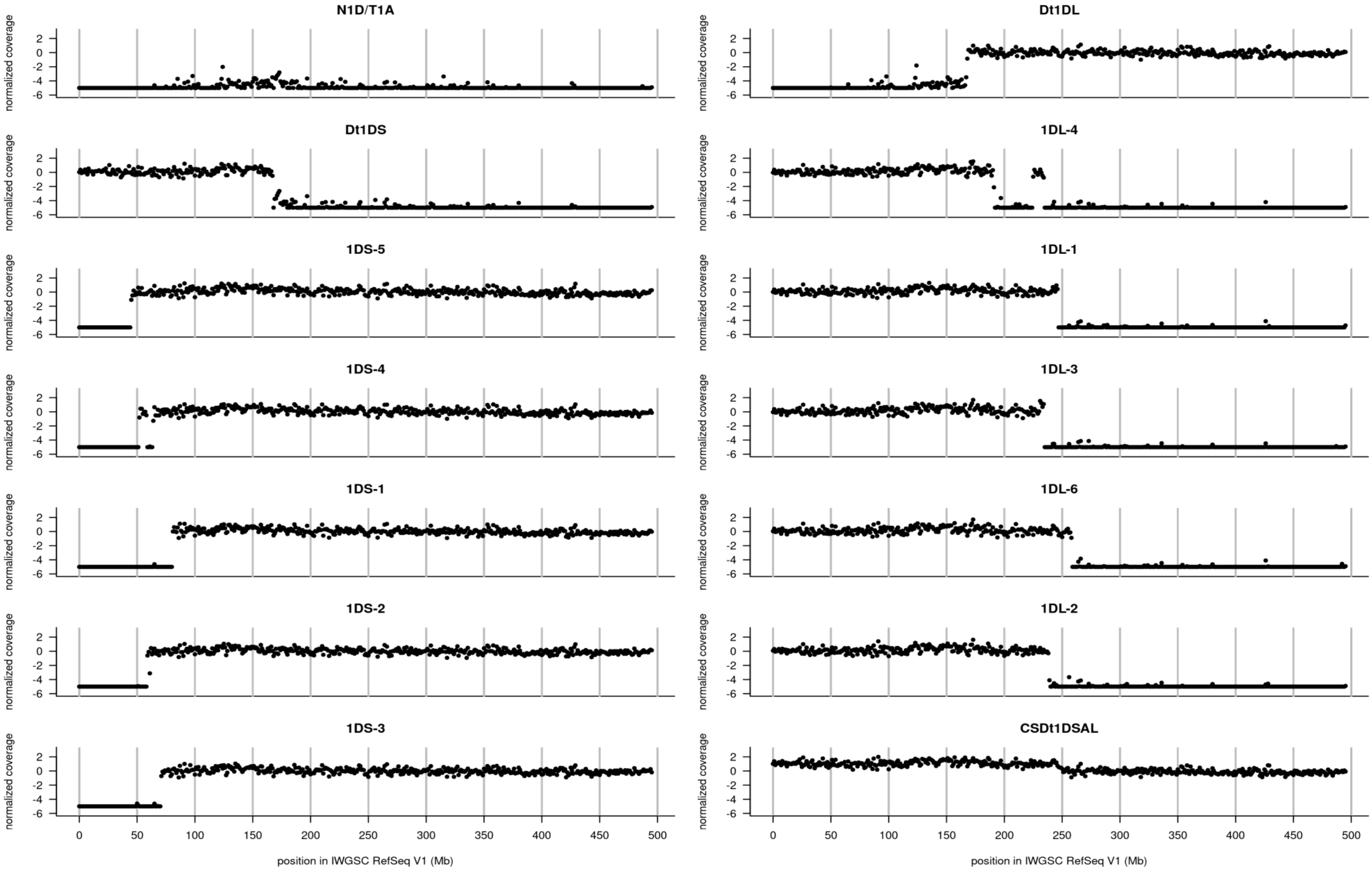

Supplementary Figure 2: Normalized read coverage in 1 Mb bins along chromosome 1D of the Chinese Spring V1.0 reference assembly. (GBS-1:0).

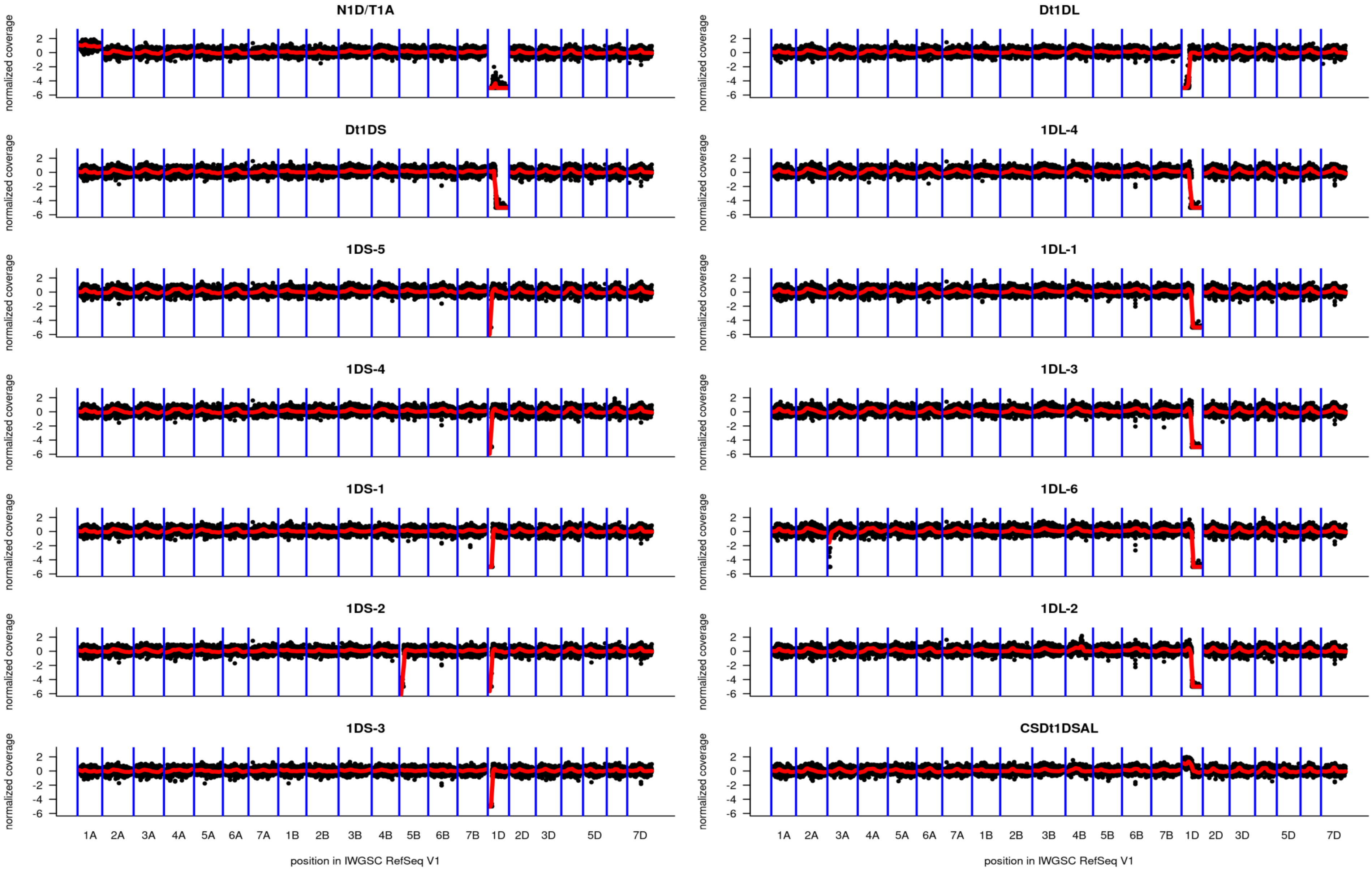

Supplementary Figure 3: Normalized read coverage in 1 Mb bins along with the Chinese Spring V1.0 reference assembly. (GBS-ALL:).

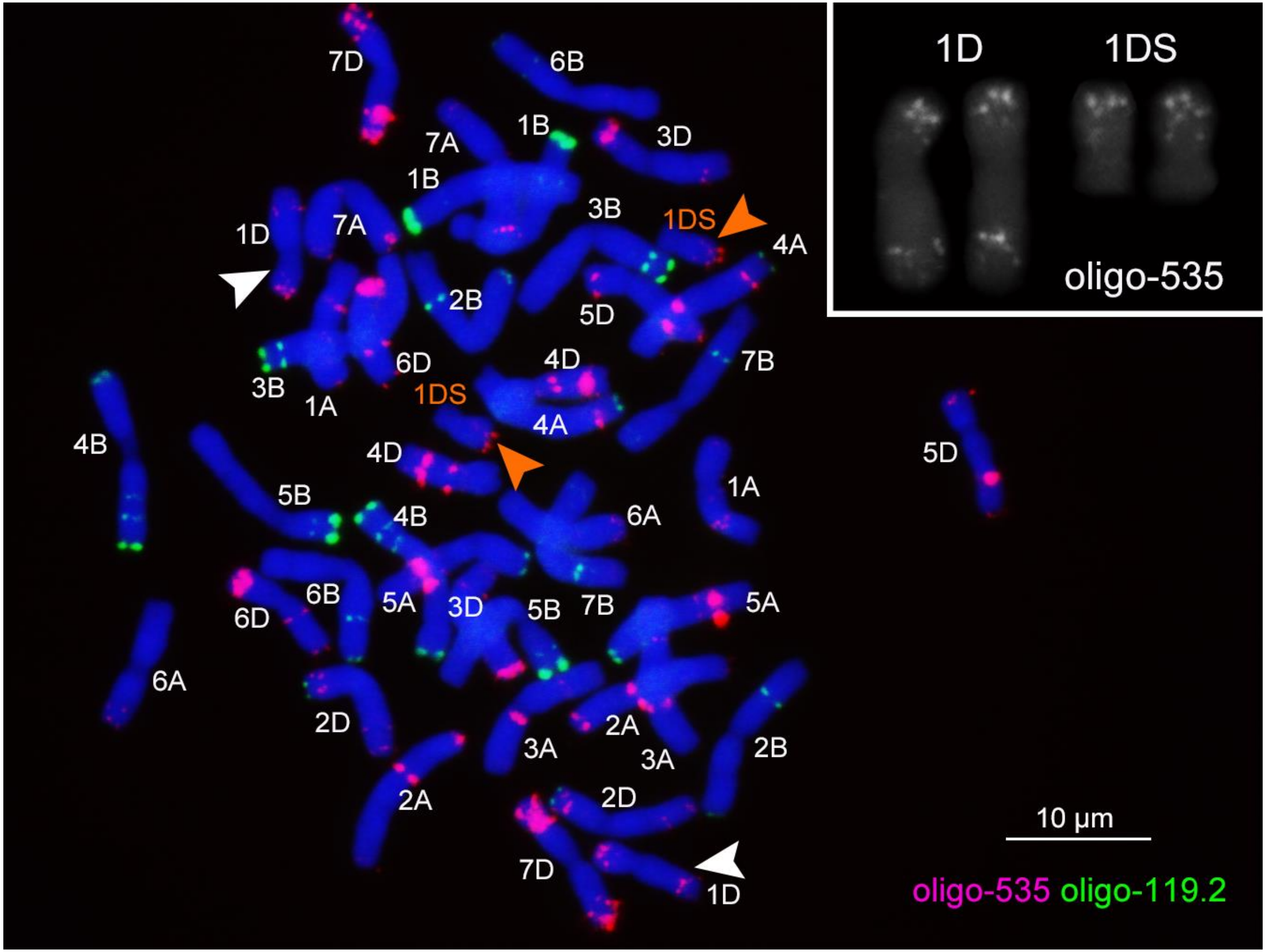

Supplementary Figure 4: FISH-based analysis of mitotic chromosomes of line 1DL-8 line using labeled oligo-119.2 (green) and oligo-535 (magenta) probes. Chromosomes were counterstained with DAPI (blue). Chromosome pairs 1D (white) and 1DS (orange) are marked with arrowheads and further enlarged after FISH exhibiting oligo-535 signals.

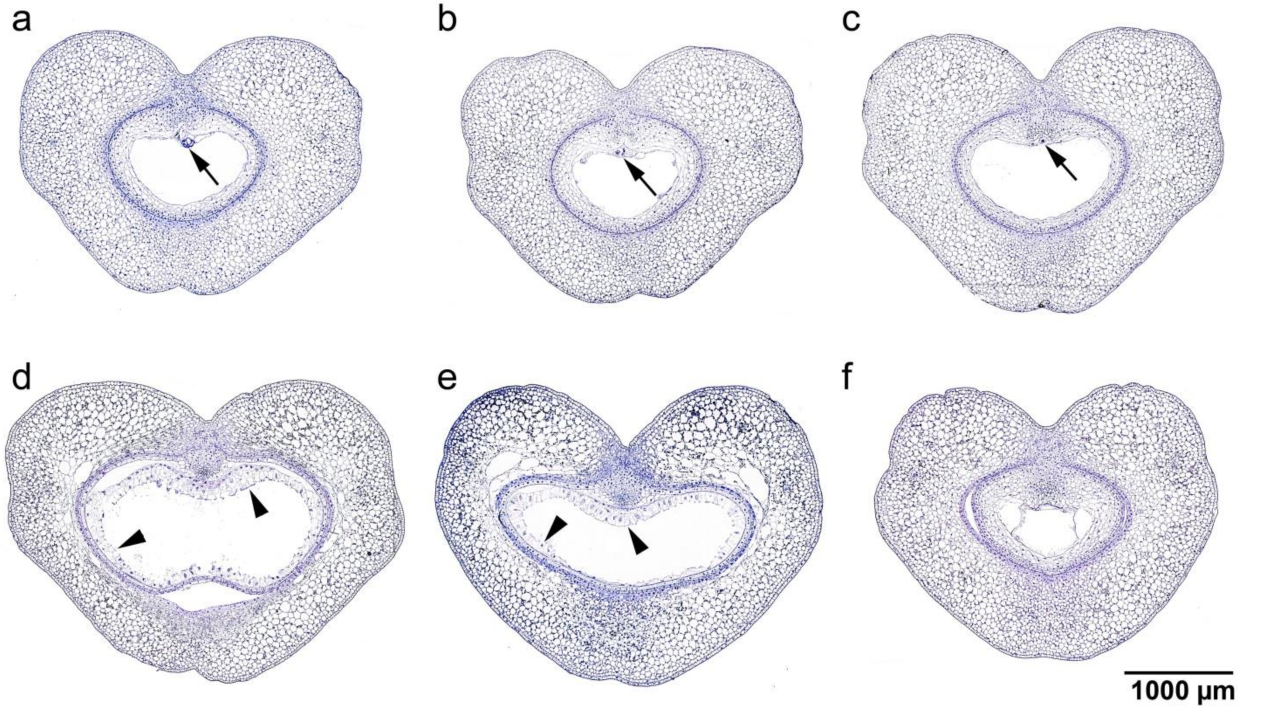

Supplementary Figure 5: Medial transverse sections through developing caryopsis at 2 DAP (a-c) and 5 DAP (d-f). At 2 DAP caryopses of CS x CS (a), CS x rye (b) and N1D/T1A x rye (c) have a similar phenotype showing a small embryogenic cavity and absence of cellularized endosperm; arrows in a-c indicate antipodal cells. At 5 DAP caryopses of CS x CS (d) and CS x rye (e) show a markedly enlarged embryogenic cavity and the initiation of endosperm cellularization (arrowheads in (d) and (e)). In the 5 DAP caryopses of line N1D/T1A (f) the embryonic cavity is not enlarged and there is no sign of endosperm cellularization. Bar = 500 µm.

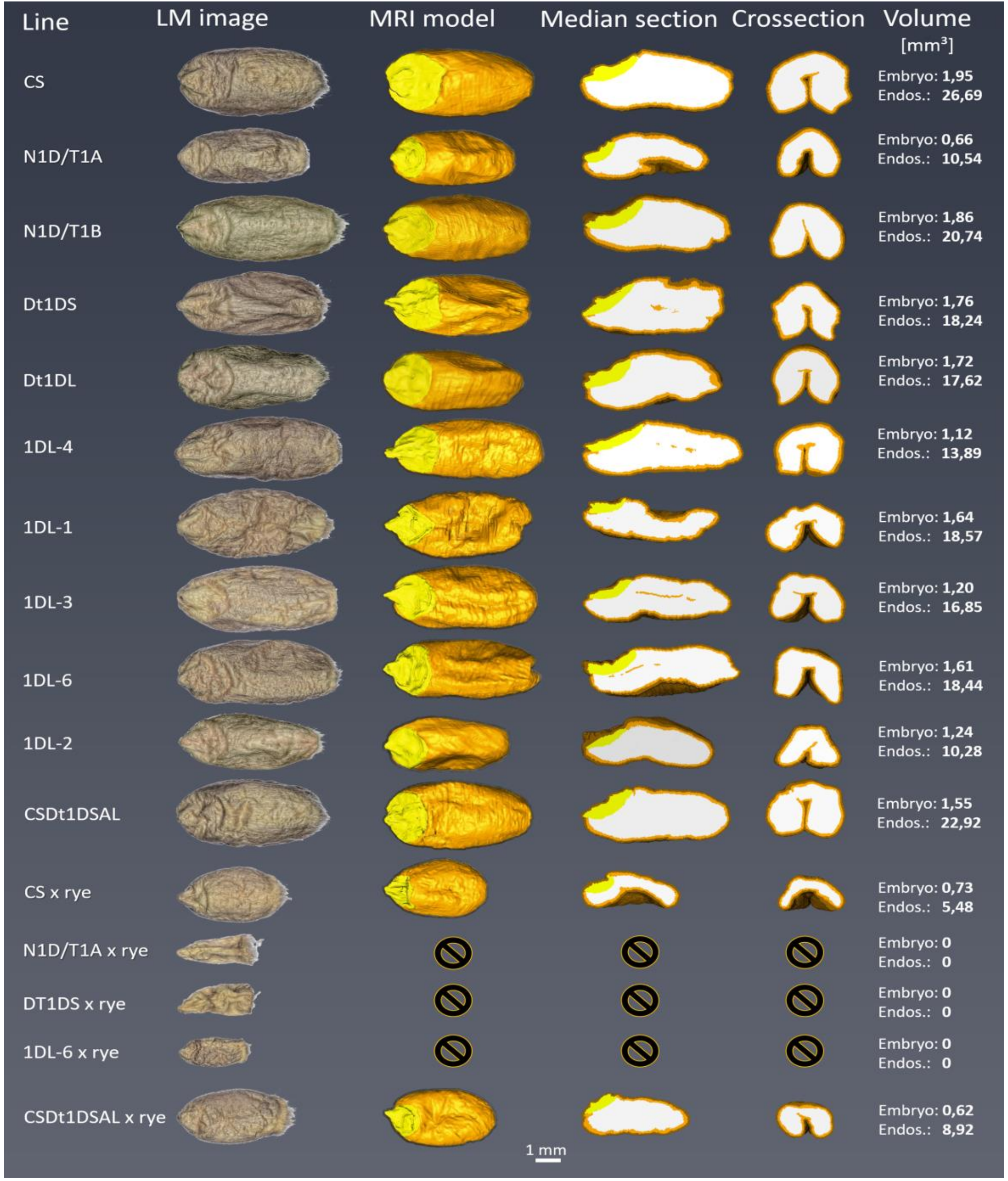

Supplementary Figure 6: Volume of embryo and endosperm at HM stage as measured by MRI of intact dry grains of representative lines and crosses.

a) 2016_GH

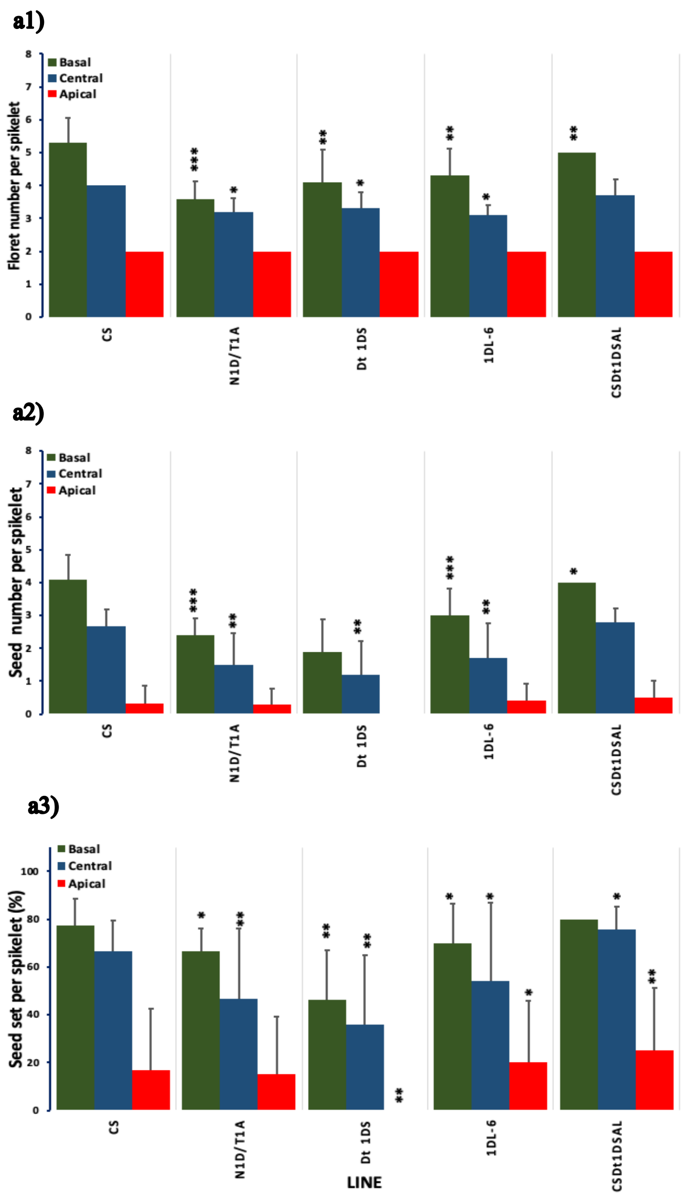

Supplementary Figure 7a: Spikelet productivity in relation to their location on the spike. (a1) floret number per spikelet; (a2) seed number per spikelet; (a3) seed fertility per spikelet. Plants were grown under greenhouse conditions in 2016 and examined during the maturity stage. n = 5-10 biologically independent samples. Data are expressed as mean ± SEM. The degree of significance indicated as *P, 0.05; **P, 0.01; ***P, 0.001; ns: not significant.

b) 2017_GH

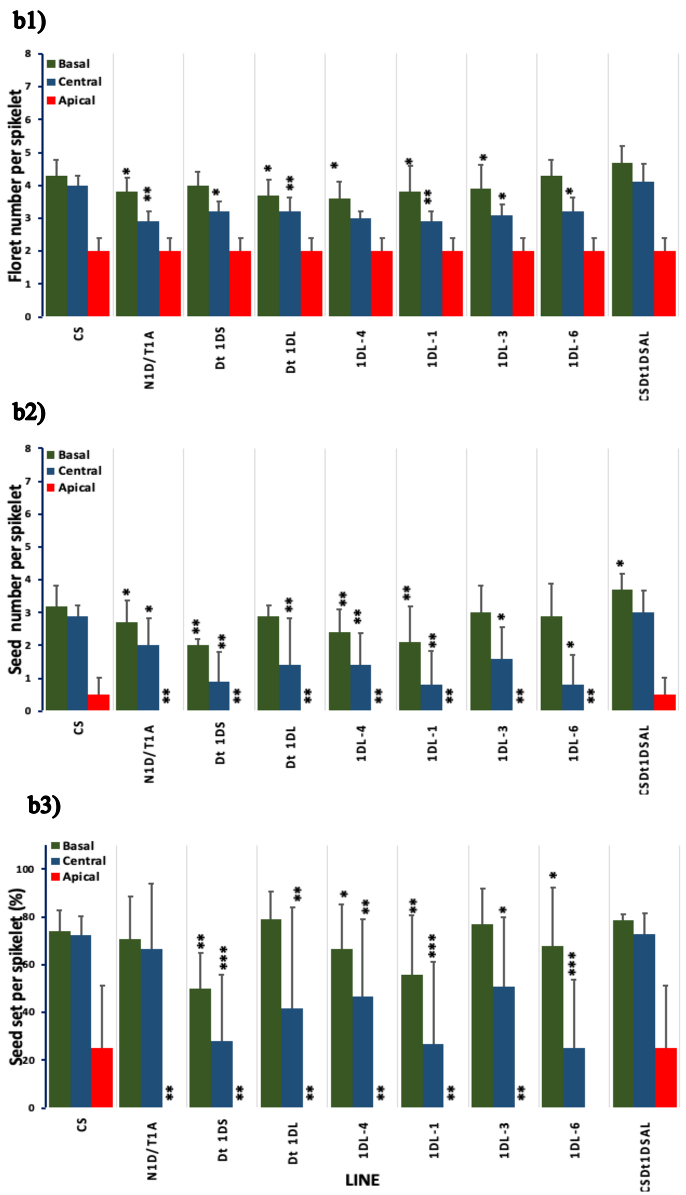

Supplementary Figure 7b: Spikelet productivity in relation to their location on the spike. (b1) floret number per spikelet; (b2) seed number per spikelet; (b3) seed fertility per spikelet. Plants were grown under greenhouse conditions (2017) and examined during the maturity stage. n = 5-10 biologically independent samples. Data are expressed as mean ± SEM. The degree of significance indicated as *P, 0.05; **P, 0.01; ***P, 0.001; ns: not significant.

c) 2018_GH

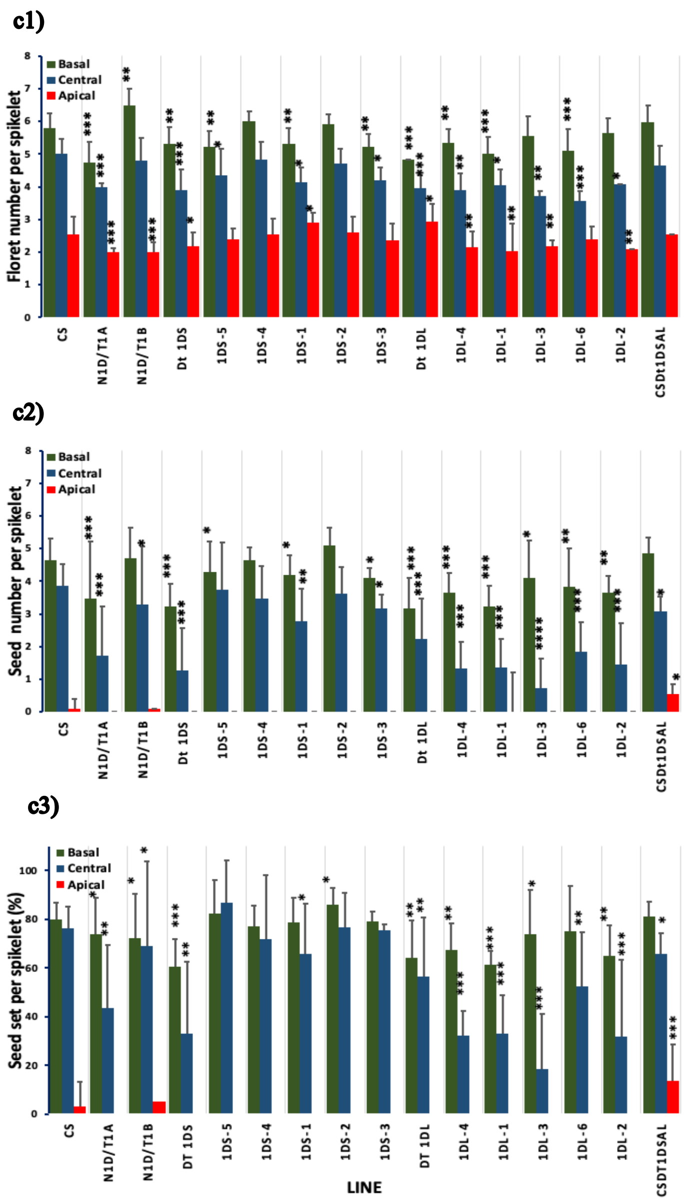

Supplementary Figure 7c: Spikelet productivity in relation to their location on the spike. (c1) floret number per spikelet; (c2) seed number per spikelet; (c3) seed fertility per spikelet. Plants were grown under greenhouse conditions (2018) and examined during the maturity stage. n = 5-10 biologically independent samples. Data are expressed as mean ± SEM. The degree of significance indicated as *P, 0.05; **P, 0.01; ***P, 0.001; ns: not significant.

d) 2018_Field

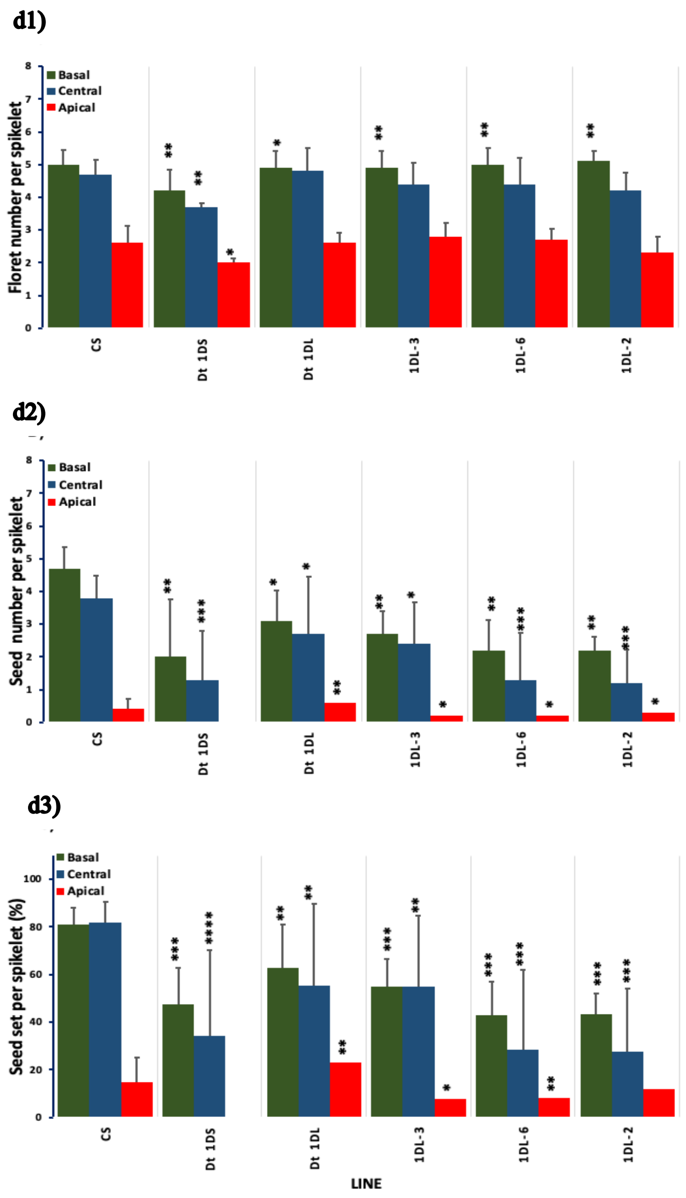

Supplementary Figure 7d: Spikelet productivity in relation to their location on the spike. (d1) floret number per spikelet; (d2) seed number per spikelet; (d3) seed fertility per spikelet. Plants were grown under greenhouse conditions (2018) and examined during the maturity stage. n = 5-10 biologically independent samples. Data are expressed as mean ± SEM. The degree of significance indicated as *P, 0.05; **P, 0.01; ***P, 0.001; ns: not significant.

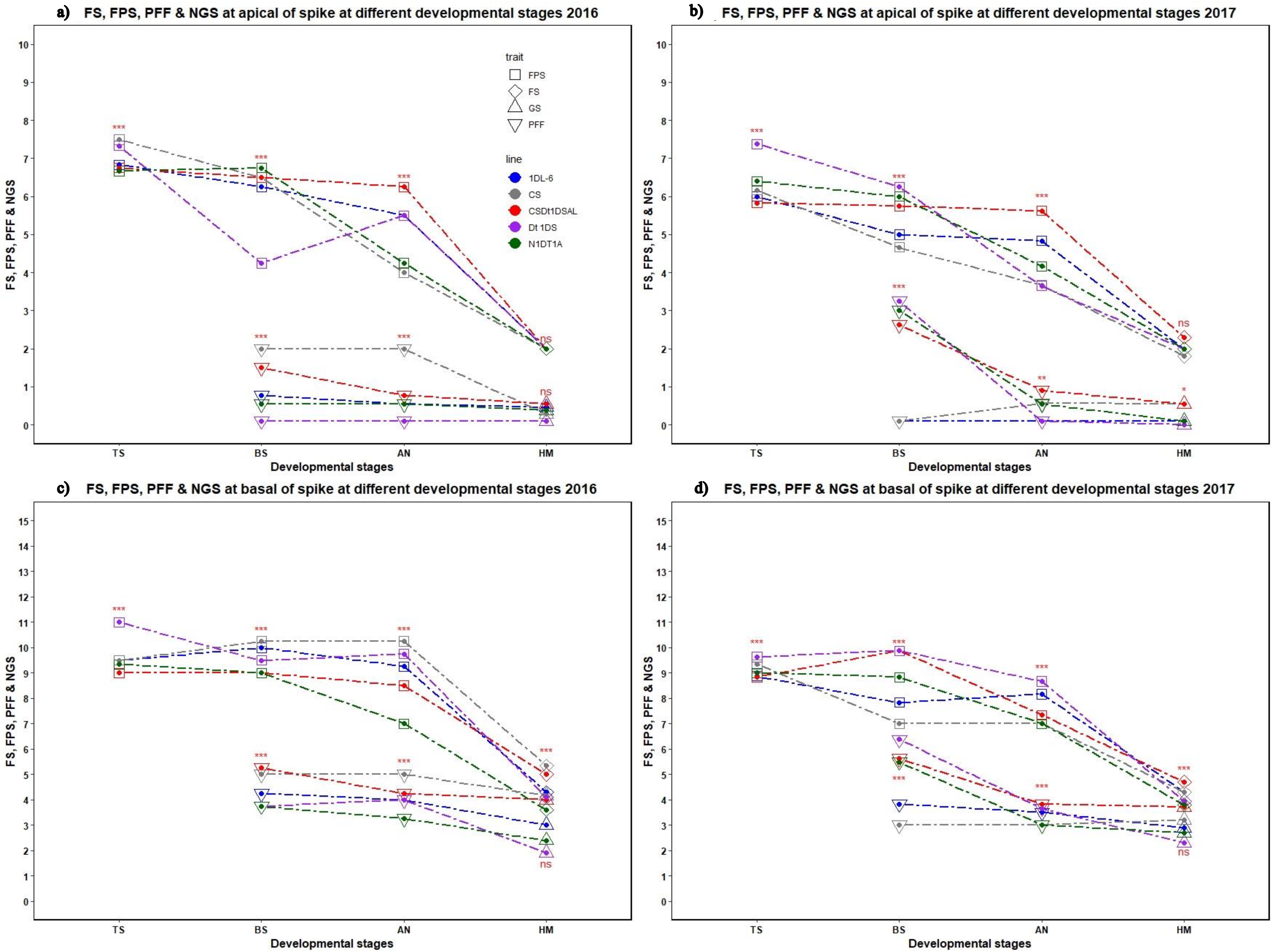
a) FS, FPS, PFF & NGS at apical of spike at different developmental stages 2016
b) FS, FPS, PFF & NGS at apical of spike at different developmental stages 2017
c) FS, FPS, PFF & NGS at basal of spike at different developmental stages 2016
d) FS, FPS, PFF & NGS at basal of spike at different developmental stages 2017
FS, FPS, PFF & NGS
Developmental stages
TS
BS
AN
HM
trait
FPS
FS
GS
PFF
line
1DL-6
CS
CSDt1DSAL
Dt 1DS
N1DT1A
***
**
*
ns

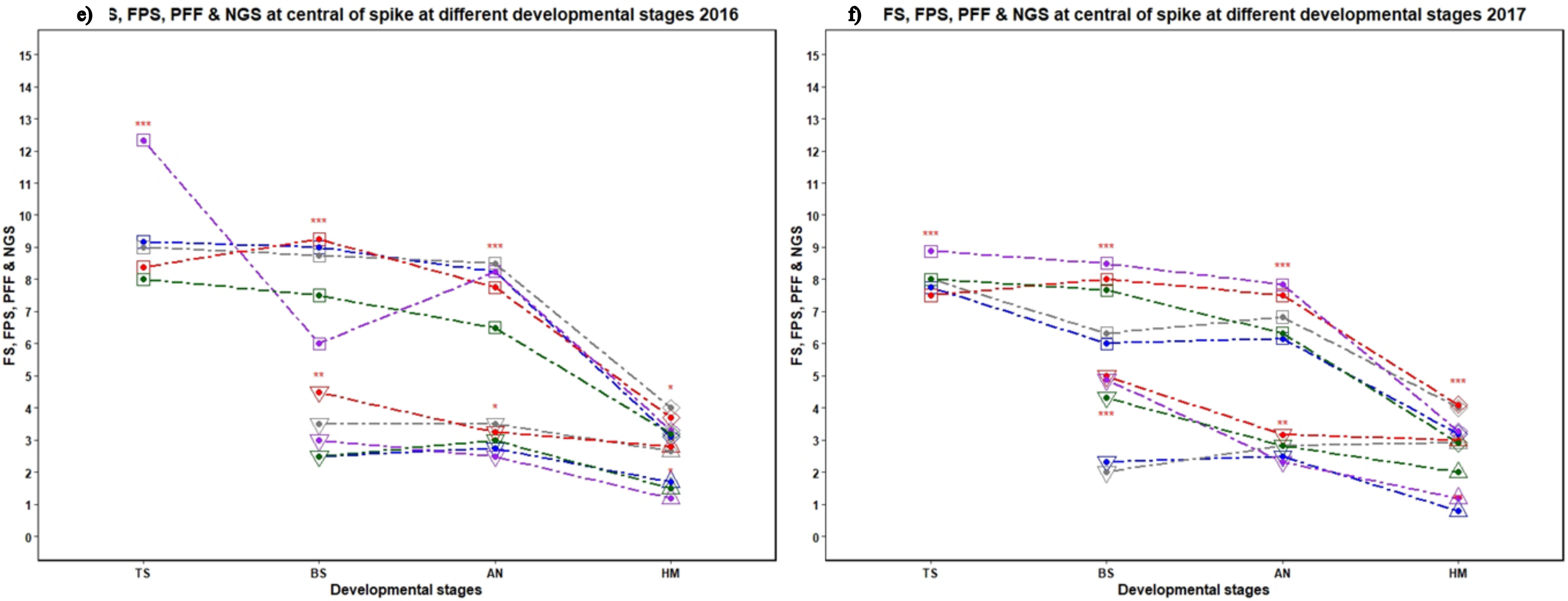

Supplementary Figure 8: Number of floret primordia per spikelet in TS and spikelet fertility in HM stages. Dee-D1 locus (loci) present in CS and CSDt1DSAL and absent in 1DL-6, N1D/T1A and Dt1DS lines. The number of floret primordia per spikelet (FPS) in TS stage, number potential fertile florets per spikelet (PFS) in BS and AN stage, floret (FS) and number grains per spikelet (NGS) in HM in apical (a, b), central (c, d) and basal (e, f) positioned spikelet along with the wheat spike. Lines were grown under greenhouse conditions in 2016 (a, c, e) and 2017 (b, d, f). n = 3 biologically independent samples. The degree of significance indicated as *P, 0.05; **P, 0.01; ***P, 0.001; ns: not significant.

Wheat or wheat line	Number of copies the *Dee* loci in cells				Endosperm development	
	in somatic cells (genotype)	in central cell in embryo sac	in endosperm cells after fertilisation		selfpollina-tion	in cross with rye
			selfpollination	in cross with rye		
Tetraploid wheat	2 *Dee-B1*	2 *Dee-B1*	3 *Dee-B1*	2 *Dee-B1* + R	normal	endosperm abbortion
Hexaploid wheat CS	2 *Dee-B1*<br>2 *Dee-D1*	2 *Dee-B1*<br>2 *Dee-D1*	3 *Dee-B1*<br>3 *Dee-D1*	2 *Dee-B1*<br>2 *Dee-D1* + R	normal	viable hybrid seeds
N1D/T1A	2 *Dee-B1*	2 *Dee-B1*	3 *Dee-B1*	2 *Dee-B1* + R	- 25-30%	endosperm abbortion
N1D/T1B	4 *Dee-B1*	4 *Dee-B1*	6 *Dee-B1*	4 *Dee-B1* + R	nornal	67% viable
Dt1DS	2 *Dee-B1*	2 *Dee-B1*	3 *Dee-B1*	2 *Dee-B1* + R	- 25-30%	endosperm abbortion
CSDt1DSAL	2 *Dee-B1*<br>2 *Dee-D1*	2 *Dee-B1*<br>2 *Dee-D1*	3 *Dee-B1*<br>3 *Dee-D1*	2 *Dee-B1*<br>2 *Dee-D1* + R	normal	viable hybrid seeds

Supplementary Figure 9: Number of copies Dee-D1 and Dee-B1 loci in different cells of tetraploid and hexaploid wheat.

Supplementary Table 1: Accession codes for genotyping-by-sequencing raw data

sample.nam	accession.ID	ENA.id	all_reads	mapped_reads	uniquely_mapped_q20
N1D/T1A	ERS4427891	ERS4427891	12,479,926	12,372,242	8,264,217
Dt1DS	ERS4427892	ERS4427892	12,553,359	12,427,106	8,152,718
1DS-5	ERS4427893	ERS4427893	12,292,143	12,176,852	7,893,371
1DS-4	ERS4427894	ERS4427894	13,421,330	13,319,036	8,733,762
1DS-1	ERS4427895	ERS4427895	12,579,739	12,473,835	8,306,682
1DS-2	ERS4427896	ERS4427896	12,613,808	12,541,593	8,432,412
1DS-3	ERS4427897	ERS4427897	8,734,442	8,635,488	5,678,839
Dt1DL	ERS4427898	ERS4427898	15,136,158	14,849,533	9,762,278
1DL-4	ERS4427899	ERS4427899	10,635,712	10,560,565	7,078,376
1DL-1	ERS4427900	ERS4427900	11,110,233	10,957,376	7,295,540
1DL-3	ERS4427901	ERS4427901	6,409,258	6,327,195	4,180,037
1DL-6	ERS4427902	ERS4427902	13,245,669	13,154,199	8,873,367
1DL-2	ERS4427903	ERS4427903	7,464,423	7,401,131	4,815,790
CSDt1DSAL	ERS4427904	ERS4427904	8,799,843	8,719,587	5,756,444

Supplementary Table 2a: Characteristics of the main spike of hexaploid wheat CS, CS N1D/T1A, N1D/T1B and the set of CS deletion lines of chromosome 1D (greenhouse 2018).

Line	Characteristics of the main spike					Breakpoint interval[1)]
	Length (cm)	Number of spikelets	Number of seeds	TGW (g)	Seed set (%)	
CS	9.7	31.6	73.8	33.0	89.5	-
N1D/T1A	8.3*	28.0*	45.4***	24.9***	64.2***	-
N1D/T1B	9.2	31.0	65.6*	26.9**	86.5	-
Dt1DS	10.2	27.0**	33.0***	25.7***	52.8***	Cen -1.0↑
1DS-5	8.6*	29.4	66.6*	42.5***	92.1	+ 0.70
1DS-4	9.4	31.4	72.8	33.7	90.0	+ 0.66
1DS-1	8.6*	29.6	57.4**	29.4*	77.6**	+ 0.59
1DS-2	9.4	28.4*	62.0**	33.9	79.6*	+ 0.57
1DS-3	9.2	30.4	57.2**	32.7	79.0*	+ 0.48
Dt1DL	7.2***	25.6***	44.4***	27.2**	67.0**	Cen - 1.0↓
1DL-4	11.9***	29.2	39.2***	34.6	54.4***	- 0.18
1DL-1	10.9**	29.6	30.6***	28.6*	47.5***	- 0.23
1DL-3	11.0**	31.2	42.8***	31.7	58.3***	- 0.25
1DL-6	11.7**	30.4	51.4***	29.1*	59.8***	- 0.29
1DL-2	11.3**	31.6	34.8***	27.0**	55.7***	- 0.41
CSDt1DSAL	8.8**	30.2	77.0	34.3	90.7	-

The degree of significance indicated as **P, 0.05*; ***P, 0.01*; ****P, 0.001*. *n* = 6 biologically independent samples.

1)- breakpoint intervals according to the data from https://www.k-state.edu/wgrc/genetic_resources/deletion_lines/group_1.html

CS: Chinese Spring, TGW: thousand-grain weight

Supplementary Table 2b: Characteristics of the main spike of hexaploid wheat CS, CS N1D/T1A, N1D/T1B and the set of CS deletion lines of chromosome 1D (field 2018)

Line	Characteristics of the main spike					Seed viability[1)]
	Length (cm)	Number of spikelets	Number of seeds	TGW (g)	Seed set (%)	
CS	8.5	24.2	53.0	27.7	72.5	+
Dt1DS	9.7**	21.6***	19.6**	19.3***	40.6***	-
Dt1DL	8.1*	21.1***	35.3**	20.6***	60.8*	+
1DL-3	10.0***	22.0***	34.7**	21.3***	58.3*	-
1DL-6	10.0***	22.4**	26.5**	19.9***	42.6**	-
1DL-2	10.3***	21.7***	21.2***	20.1***	37.4***	-

The degree of significance indicated as **P, 0.05*; ***P, 0.01*; ****P, 0.001*. *n* = 10 biologically independent samples.

1) viable (+) and non-viable (-) hybrid seeds in cross with rye; CS: Chinese Spring, TGW: thousand-grain weight

Supplementary Table 2c: Characteristics of the main spike of hexaploid wheat CS, CS N1D/T1A, Dt 1DS and the set of CS deletion lines of chromosome 1D (greenhouse, 2016)

Line	Characteristics of the main spike					Seed viability[1)]
	Length (cm)	Number of spikelets	Number of seeds	TGW (g)	Seed set (%)	
CS	7.2	26.0	47.0	32.5	86.8	+
N1D/T1A	7.9	28.8*	30.4*	15.0***	65.1**	-
Dt1DS	9.2***	25.8**	18.6***	21.7**	38.9***	-
1DL-6	10.2**	27.6	39.6*	27.6	58.7**	-
CSDt1DSAL	7.8	29.6*	62.0**	30.6	91.0	+

The degree of significance indicated as **P, 0.05*; ***P, 0.01*; ****P, 0.001*. $n = 10$ biologically independent samples.

•viable (+) and non-viable (-) hybrid seeds in cross with rye;

CS: Chinese Spring, TGW: thousand-grain weight.

Supplementary Table 2d: Characteristics of the main spike of hexaploid wheat CS, CS N1D/T1A, Dt 1DS and the set of CS deletion lines of chromosome 1D (greenhouse, 2017).

Line	Characteristics of the main spike					Seed viability[1)]
	Length (cm)	Number of spikelets	Number of seeds	TGW (g)	Seed set (%)	
CS	7.9	25.2	45.8	29.3	84.8	+
N1D/T1A	7.6*	31.4***	39.2*	22.7**	75.4*	-
Dt1DS	10.1***	27.2*	24.2**	25.0*	45,7***	-
Dt1DL	6.0***	24.2	25.0**	23.1**	58.4**	+
1DL-4	9.8***	24.6	24.8**	28.3	50.0***	-
1DL-1	9.3**	26.6	21.4***	24.5**	48.6***	-
1DL-3	11.2***	25.2	28.4**	21.3**	46.7***	-
1DL-6	10.3***	26.4	29.8**	21.5*	51.3**	-
CSDt1DSAL	8.5*	28.2**	63.0***	30.6	87.7	+

The degree of significance indicated as **P, 0.05*; ***P, 0.01*; ****P, 0.001*. *n* = 8 biologically independent samples.

•viable (+) and non-viable (-) hybrid seeds in cross with rye;

CS: Chinese Spring, TGW: thousand-grain weight.

Supplementary Table 3: Implementation of potential productivity in the spikes of hexaploid wheat CS and in CS N1D/T1A, Dt 1DS, 1DL-6, CSDt1DSAL lines (greenhouse 2016, 2017).

Year	Line	Mean number of flowering meristems and grains in different parts of the spike								
		basal			central			apical		
		FPS[1)]	NGS[2)]	%[3)]	FPS	NGS	%	FPS	NGS	%
2016	CS	9.5	4.2	44.2	9.0	2.7	30.0	7.5	0.3	4.0
	N1D/T1A	9.3*	2.4**	25.8***	8.0	1.5*	18.8**	6.7*	0.3	4.5
	Dt 1DS	11.0 **	2.3**	20.1***	12.3**	1.0**	8.1***	7.3	0.0*	0.0**
	1DL-6	9.5	3.0*	31.6**	9.2	1.7*	18.5**	6.8*	0.4	5.9*
	CSDt1DSAL	8.8*	4.0	45.5	8.7	2.8	32.2	7.0	0.8**	11.4***
2017	CS	9.3	3.1	33.3	8.0	2.9	36.3	7.5	0.3	4.0
	N1D/T1A	9.1	2.7	29.7	7.9	2.0*	25.3*	6.7*	0.0*	0.0*
	Dt 1DS	9.6*	2.3*	24.0**	8.9**	1.2***	13.5***	7.3	0.0*	0.0*
	1DL-6	8.8**	2.3*	26.1	7.9	0.8***	10.1***	6.8*	0.0*	0.0*
	CSDt1DSAL	8.8**	3.7*	42.0	7.5*	3.0	40.0	7.0	0.5*	7.1**

[1)] – number floret primordia per spikelet (FPS) in terminal spikelet (TS) stage; [2)] – number grains per spikelet (NGS) in harvest maturity (HM) stage;
[3)] – percent of floret meristem form the grains;
The degree of significance indicated as **P, 0.05*; ***P, 0.01*; ****P, 0.001*. n = 3 biological independent samples.
