# Supplementary material for: DEFECTIVE ENDOSPERM-D1 (Dee-D1) is crucial for endosperm development in hexaploid wheat
Source: Commun Biol. 2020 Dec 23;3:791. doi: 10.1038/s42003-020-01509-9 (PMC7758331; doi:10.1038/s42003-020-01509-9)
Supplement: Supplementary file 3 — Description of Additional Supplementary Files [file 42003_2020_1509_MOESM3_ESM.pdf]

Description of Additional Supplementary Files

File Name: Supplementary Data 1
Description: Detailed phenotypic data of the main spike characteristics in hexaploid wheat under different growing conditions.

File Name: Supplementary Data 2
Description: The list of candidate genes located within the physical deletion interval in the CS wheat genome.

File Name: Supplementary Movie 1
Description: Shape and volume of endosperm as revealed by comparative 3DMRI analysis of intact mature grains of representative lines and crosses
